# Neural and somatic mechanisms driving clinical improvements in post-acute schizophrenia spectrum disorders

**DOI:** 10.1192/j.eurpsy.2025.10129

**Published:** 2025-10-27

**Authors:** Lukas Roell, Christoph Lindner, Isabel Maurus, Daniel Keeser, Berend Malchow, Andrea Schmitt, Peter Falkai

**Affiliations:** 1 Department of Psychiatry and Psychotherapy, LMU University Hospital, Ludwig-Maximilians-University Munich, Munich, Germany; 2 Max Planck Institute of Psychiatry, Munich, Germany; 3 NeuroImaging Core Unit Munich (NICUM), LMU University Hospital, Ludwig-Maximilians-University Munich, Munich, Germany; 4 Department of Psychiatry and Psychotherapy, University Medical Center Göttingen, Göttingen, Germany; 5 Laboratory of Neuroscience (LIM27), Institute of Psychiatry, University of Sao Paulo, São Paulo, Brazil; 6 German Center for Mental Health (DZPG), Partner Site Munich-Augsburg, Munich, Germany

**Keywords:** schizophrenia, clinical trajectories, MRI, gray matter volume, exercise

## Abstract

**Background:**

A better mechanistic understanding of schizophrenia spectrum disorders is crucial to developing efficient treatment approaches. Therefore, this study investigated longitudinal interrelations between clinical outcomes, brain structure, and somatic health in post-acute individuals from the schizophrenia spectrum.

**Methods:**

A sample of 63 post-acute patients from two independent physical exercise studies was included in the final analyses. Demographic, clinical, cognitive, and somatic data were acquired at baseline and follow-up, as were structural magnetic resonance imaging scans. Multivariate cross-lagged panel modeling including mediators was used to study the mutual interrelations over time between the clinical, neural, and somatic levels.

**Results:**

A higher baseline global gray matter volume and larger regional gray matter volumes of the hippocampal formation, precuneus, and posterior cingulate predicted improved clinical outcomes, such as daily-life functioning, negative symptoms, and cognition. Increases in white matter volume from baseline to follow-up resulted in significantly reduced positive symptoms and higher daily-life functioning.

**Conclusions:**

Our findings suggest that stimulating neuroplasticity, especially in the hippocampal formation, precuneus, and posterior cingulate gyrus, may represent a promising treatment target in post-acute schizophrenia spectrum disorders. Physical exercise therapies and other lifestyle interventions, and brain stimulation approaches reflect potential treatment candidates. Given the exploratory character of the statistical analysis performed, these findings need to be replicated in independent longitudinal imaging cohorts of patients with schizophrenia spectrum disorders.

## Introduction

Schizophrenia spectrum disorders (SSD) are debilitating psychiatric conditions characterized by positive, negative, and cognitive symptoms, as well as by long-term impairments in daily life functioning in a significant proportion of patients [1]. Apart from those behavioral domains, patients with SSD are often affected by substantial somatic comorbidities, resulting in significant reductions in general body and organ health [2]. Current treatment options encompass antipsychotic medication, psychotherapy, cognitive remediation, non-invasive brain stimulation, or physical exercise interventions, each differing with regard to their therapeutic windows and the underlying treatment goals [3–7]. Despite the efficiency of these interventions, the current long-term disease outcomes in people with SSD offer substantial room for improvement: For instance, current estimates on remission rates averaged across different patient populations hover between 26 and 58% [8, 9], whereas recovery rates only range between 13.5 and 36% [8–11]. This places SSD among the top 20 diseases with the highest Years-Lived-With-Disability index [12], emphasizing the urgent need to improve current treatment options.

To enhance the long-term therapeutic success in SSD, a better mechanistic understanding of clinical symptoms, cognitive deficits, and daily-life functioning is warranted. Such mechanisms in SSD may be observable on the cerebral level assessed via structural magnetic resonance imaging (MRI). Large-scale cross-sectional evidence demonstrates that structural deteriorations in key brain regions such as the insula, anterior and posterior cingulate cortex, superior and middle frontal cortices, precuneus, hippocampal formation, putamen, pallidum, caudate, thalamus, and amygdala, or the cerebellum are linked to different domains of psychiatric symptoms and cognitive functioning in SSD [13–22]. Moreover, widespread alterations in white matter integrity have been associated with cognitive deficits [23]. However, these brain-behavior associations obtained from cross-sectional data yield only limited pathophysiological relevance, as they do not allow for making inferences on how the neural level affects the clinical level over time and vice versa.

To address this issue, longitudinal neuroimaging studies are warranted. Respective evidence reveals an accelerated gray matter volume loss over time across multiple brain regions in SSD linked to the type of antipsychotic treatment and therefore potentially also to the long-term course of symptom severity [24–26]. In line with these findings, gray matter volumes can serve as a predictor of long-term clinical outcomes in SSD [27], although many machine learning studies in this field have noticeable shortcomings, such as a limited generalizability due to small sample sizes [28, 29]. In addition to methodological concerns, the exact mechanisms of action between brain structures and specific symptom domains in SSD remain largely unknown.

Besides the neural level, the significance of deteriorations in somatic health in SSD and other psychiatric conditions has been increasingly noticed in recent years [2]. For instance, obesity in SSD is linked to both overall symptom severity [30] and impaired brain structure [31]. However, the longitudinal interrelations between somatic health, clinical domains, and brain structure need to be further investigated in people with SSD.

Hence, with an emphasis on SSD-relevant brain structures supported by large-scale evidence, we adopt an exploratory research approach, drawing on longitudinal data from two exercise intervention studies in SSD. First, we investigate the temporal interrelations among symptom severity, cognition, daily-life functioning, brain volumes, and somatic health in post-acute patients with SSD. Second, we examine whether changes in brain volumes and somatic health over time mediate the reciprocal associations between symptom severity, cognitive performance, and daily-life functioning. Importantly, although our data were derived from two exercise trials, our aim was not to study exercise-specific effects. Instead, we focused on the broader interrelations between clinical, somatic, and structural brain variables in post-acute SSD across different treatment contexts.

## Methods

This work utilizes data from two physical exercise studies conducted in people with SSD. One is the Enhancing Schizophrenia Prevention and Recovery through Innovative Treatments (ESPRIT) C3 study (NCT03466112) performed at different sites across Germany [32, 33]. In the project at hand, only data acquired at the Department of Psychiatry and Psychotherapy of the Ludwig-Maximilians-University Hospital in Munich were utilized. The second exercise trial (NCT01776112) was executed at the Department of Psychiatry and Psychotherapy of the University Medical Center Goettingen [34, 35].

Both studies were in line with the Declaration of Helsinki and ethical approval was provided by the local ethics committees of the Ludwig-Maximilians-University Hospital and the University Medical Center Goettingen, respectively.

### Sample and study design

The ESPRIT C3 study investigated the effects of two different types of exercise on several health outcomes in people with SSD. Patients were either randomized to an aerobic exercise intervention on bicycle ergometers or to a flexibility, strengthening, and balance training. Both groups exercised three times per week for between 40 and 50 minutes over a period of six months. For this work, we considered behavioral and MRI data from baseline and follow-up. Details on the study design and the main results are described elsewhere [32, 33].

The second study entailed a three-month aerobic exercise intervention on bicycle ergometers with additional cognitive remediation starting in the sixth week of the intervention. Patients exercised three times per week for 30 minutes per session. The control group played table soccer for the same amount of time and also received cognitive remediation after six weeks of the intervention. The project at hand considers behavioral and MRI data from baseline and follow-up only of the patients diagnosed with SSD.

Inclusion criteria comprised a participant´s age between 18 and 65 years, a total Positive and Negative Syndrome Scale (PANSS) score less than or equal to 75, and treatment with up to two antipsychotics. Exclusion criteria were severe neurological comorbidities, pregnancy, or suicidality. Details are provided in the original publications [32–35]. In the ESPRIT C3 study, only a subgroup of patients underwent the MRI assessments. After quality control of behavioral and MRI data (for details, see Supplementary Material), 63 people with SSD were included in the final analysis.

### MRI data acquisition

Participants of the ESPRIT C3 study were scanned in a 3 T Siemens Magnetom Skyra MRI scanner (SIEMENS Healthineers AG, Erlangen, Germany) at the Department of Radiology of the Ludwig-Maximilians-University Hospital Munich. A 3D T1-weighted magnetization-prepared rapid gradient echo (MPRAGE) sequence with an isotropic spatial resolution of 0.8 × 0.8 × 0.8 mm^3^ was acquired. In the second exercise study, a 3 T Magnetom TIM Trio MRI scanner (SIEMENS Healthineers AG, Erlangen, Germany) was used to acquire a 3D T1-weighted MPRAGE sequence at 1.0 × 1.0 × 1.0 mm^3^ isotropic resolution. Supplementary Table S1 summarizes the scanning parameters.

### Quality control and MRI data processing

All available structural images were inspected visually to evaluate the data quality. The automated quality control software MRIQC was used to compute relevant image quality metrics [36]. Further details on the quality control procedure are provided in the Supplementary Material.

Structural images were processed using the Neuromodulation and Multimodal Neuroimaging Software (NAMNIs) version 0.3 [37]. NAMNIs is mainly based on tools from FSL [38] and includes the following processing steps for structural MRI data: image reorientation to standard space, brain extraction, creation of a binary masks, linear and non-linear registration, inversion of the transformation and deformation field, brain segmentation, computation of global gray matter volume, white matter volume and cerebrospinal fluid, mapping of brain atlas of choice into the native space, and calculation of regional gray and white matter volumes.

**Figure 1.**
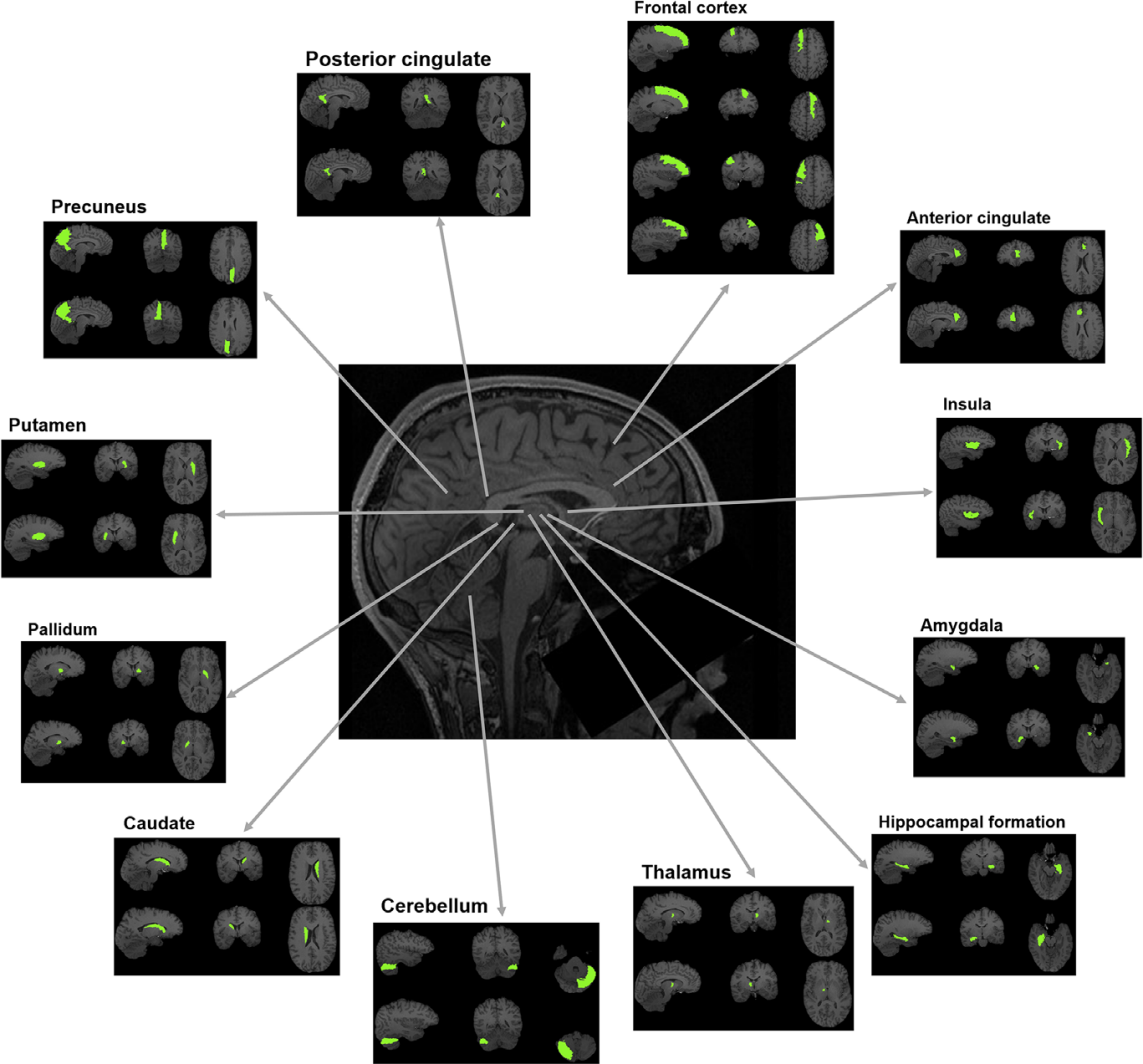
Regions of interest.

We used the third version of the automated anatomical labeling (AAL3) atlas [39] to compute the regional gray matter volumes of the bilateral insula, anterior and posterior cingulate cortices, superior and middle frontal cortices, precuneus, hippocampus, putamen, pallidum, caudate, thalamus, and amygdala. The aforementioned regions were selected based on previous literature suggesting their clinical relevance in SSD (see introduction). The regions of interest are visualized in Figure 1.

The regions of interest are visualized for which gray matter volumes were computed. Note that for the thalamus, cerebellum, and anterior cingulate gyrus only one subregion is illustrated in this figure. For the analyses, the volumes of these subregions were summed up to one volume score.

### Clinical and cognitive assessments

To assess positive and negative symptoms, the Positive and Negative Syndrome Scale (PANSS) [40] was utilized in both exercise studies, as was the Global Assessment of Functioning (GAF) scale to assess daily life functioning [41]. The first trial and the interference trial of the Verbal Learning and Memory Test (VLMT) [42] were averaged to a short-term memory score, whereas the sixth and seventh trials were averaged to a long-term memory score. The backward version of the Digit Span Test (DST) [43] was used to assess working memory. The Trail Making Tests (TMT) A and B [44] were averaged to provide a more global measure of cognition covering multiple domains such as processing speed, working memory updating, and response inhibition. A detailed description of the cognitive test batteries is provided in the Supplementary Material. Clinical assessments and cognitive tests were administered at baseline and follow-up in both exercise studies.

### Somatic health assessments

To quantify general somatic health, we computed a principal component analysis across subjects and sessions, including body-mass-index (BMI), and levels of cholesterol, HbA1c, and triglycerides as variables. We used the score on the first principal component of each subject in each session as an indicator of general health. A lower score on the first principal component was associated with a higher BMI and higher levels of cholesterol, HbA1c, and triglycerides, thus indicating a worse somatic health (Supplementary Material).

### Statistical data analysis

**Figure 2.**
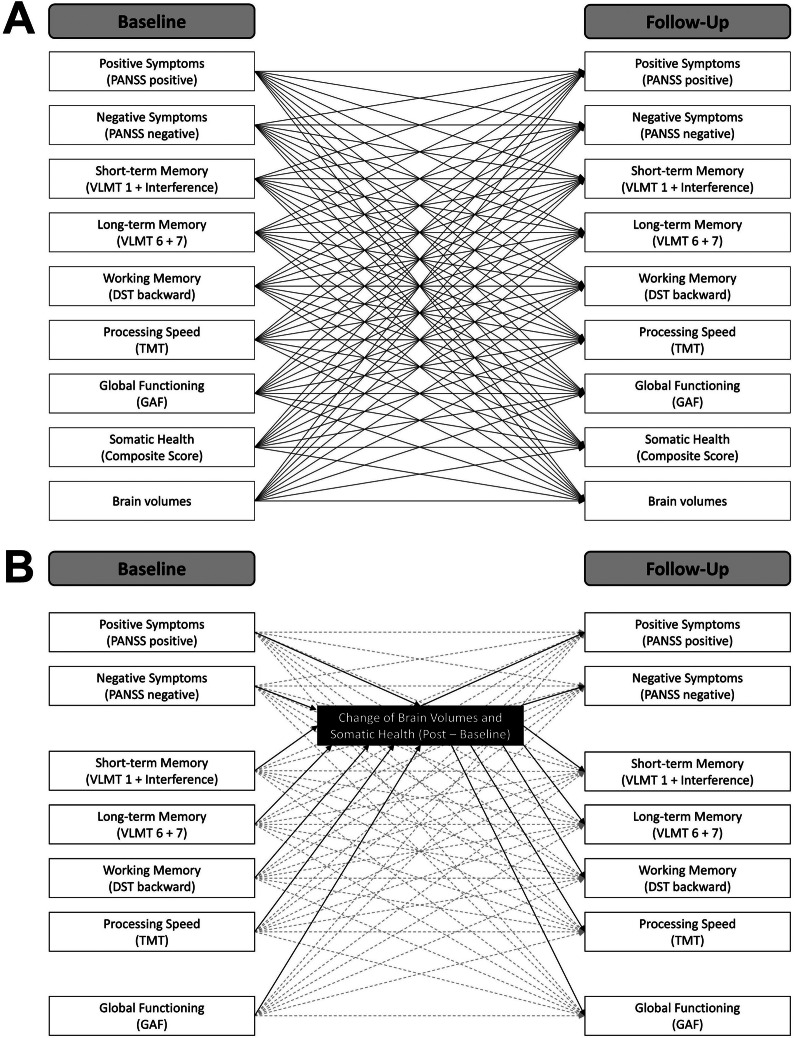
Cross-lagged panel models.

To achieve the first aim of the study, namely studying the reciprocal temporal relations between symptom severity, cognition, daily life functioning, brain volumes, and somatic health measured at baseline (first measurement occasion) and at follow-up after the intervention (second measurement occasion), multivariate cross-lagged panel modeling (CLPM) [45] was applied using the lavaan package [46] in R version 4.2.2. We fitted a total of 28 separate models for global gray and white matter volume, as well as for 13 brain regions of interest for both hemispheres. In all our models, (residual) correlations between variables at the same levels (i.e. baseline and follow-up) were allowed. Further, the models comprised exclusively manifest variables as indicators, represented in positive and negative symptom severity scores, short-term, long-term, and working memory performance scores, daily life functioning scores, somatic health scores, and the respective brain volume scores, each at baseline and follow-up. In all CLPM, the autoregressive paths represented the stability of individual differences from the first to the second measurement occasion. These are the effects of all our measured variables on themselves. The cross-lagged paths are the effects of each variable on each other variable measured at a later timepoint, while controlling for the prior level of the corresponding variable being predicted [47]. Thus, the CLPM allowed us to explore the directional interrelations between all variables of investigation over the course of time. Given the exploratory approach of this study, no correction for multiple comparisons was performed, but significant results of single brain regions were only further interpreted if they were consistent across hemispheres. Figure 2A visualizes the general structure of the cross-lagged panel models.

Following the second aim of the study, examining the potential mediating role of brain volume and somatic health changes from baseline to follow-up, we included additional manifest mediator variables in our CLPM. More precisely, we specified the CLPM as described above and additionally included the change of brain volumes and somatic health scores (i.e., follow-up – baseline) as mediators. We defined a total of 29 separate models for the change in somatic health score, global gray and white matter volume, as well as for changes in 13 brain regions of interest. Besides the two mediator variables, the models contained positive and negative symptom severity scores, short-term, long-term, and working memory performance scores, and daily life functioning scores at baseline and follow-up. Specifying CLPM with additional mediator variables allowed us to investigate the role of changes in brain volume and somatic health in the relation between baseline and follow-up levels of the investigated variables while simultaneously controlling for any additional effects of the corresponding variables in the models. No correction for multiple comparisons was performed. Significant results in the case of single brain regions were only considered if they were consistent across hemispheres. Figure 2B illustrates the general structure of the mediation models.

In all our models, age, sex (female = 0, male = 1), chlorpromazine equivalents (Defined Daily Doses method [48]), years of education, exercise group (first dummy coding: aerobic exercise = 0, flexibility, strengthening, and balance training = 1; second dummy coding: aerobic exercise = 0, table soccer = 1), and study (Goettingen = 0, Munich = 1) were included as covariates to control for confounding influences of these variables on the interrelations between the main variables of investigation. Furthermore, if not otherwise described, the relations between all variables and covariates were allowed to vary freely in all models. Thus, all our models were saturated with *df* = 0, meaning that the models had as many freely estimated parameters as observations in the data set (i.e., variances, covariances, means). The maximum likelihood estimator was used and confidence intervals were calculated. The analysis dataset was free of missing data.

The manifest path models computed in the current study are illustrated. A) Cross-lagged panel models including baseline and follow-up scores. The global and regional brain volumes were inserted separately. Note that covariances between baseline and between follow-up variables and covariates are not displayed for the sake of visibility. B) Cross-lagged panel models with mediators including clinical and cognitive variables at baseline and follow-up, as well as brain volumes and somatic health as change scores (follow-up – baseline). Autoregressive and cross-lagged paths are illustrated in dashed gray lines. Note that covariances between baseline and between follow-up scores are not shown for the sake of visibility.

## Results

### Patients characteristics

Table 1 summarizes the sample characteristics. The majority of subjects received aerobic exercise therapy. More males than females were included. Overall, the sample was at a rather post-acute phase of the disease, as indicated by relatively low positive symptom severity and average functioning scores.

**Table 1.** Sample characteristics

	Patients with SSD *N* = 63 (*n*/mean ± SD)
**Study**	
Goettingen	42
Munich	21
**Study arm**	
Aerobic exercise	27
Flexibility, strengthening, and balance training	15
Table soccer	21
**Sex**	
Female	19
Male	44
**PANSS at baseline**	
Positive symptoms	12.50 ± 5.60
Negative symptoms	16.30 ± 8.20
**GAF at baseline**	61.9 ± 11.0
**Chlorpromazine equivalents**	526 ± 524
**Education** (years)	15.4 ± 3.92
**Total number of trainings**	33.7 ± 9.20

Abbreviations: GAF, Global Assessment of Functioning Scale; *N*, total sample size; *n*, sample size per category; PANSS, Positive and Negative Syndrome Scale; SD, standard deviation.

*Note*: The sample size refers to the number of participants that were considered in the final statistical data analysis.

### Reciprocal longitudinal interrelations among clinical, neural, and somatic outcomes

Across all 28 cross-lagged panel models, multiple significant paths were detected: All autoregressive paths were significant (Supplementary Table S3). With regard to the cross-lagged paths, a higher global gray matter volume predicted higher working memory performance at follow-up (*β* = 0.36, CI = [0.09, 0.63], *p* = 0.010), whereas global white matter volume had no effect. With regard to the single brain regions, a higher bilateral hippocampal gray matter volume at baseline drove higher daily life functioning at follow-up (left: *β* = 0.21, CI = [0.04, 0.39], *p* = 0.018; right: *β* = 0.19, CI = [0.01, 0.38], *p* = 0.042). Larger gray matter volume in the bilateral precuneus resulted in lower negative symptom severity (left: *β* = −0.26, CI = [−0.09, −0.42], *p* = 0.002; right: *β* = −0.19, CI = [−0.02, −0.36], *p* = 0.027), higher working memory performance (left: *β* = 0.28, CI = [0.04, 0.51], *p* = 0.019; right: *β* = 0.35, CI = [0.13, 0.58], *p* = 0.002), and higher somatic health (left: *β* = 0.22, CI = [0.04, 0.40], *p* = 0.017; right: *β* = 0.23, CI = [0.05, 0.41], *p* = 0.011) at follow-up. Larger gray matter volume in the posterior cingulate gyrus predicted higher long-term memory performance at follow-up (left: *β* = 0.18, CI = [0.03, 0.33], *p* = 0.021; right: *β* = 0.20, CI = [0.06, 0.34], *p* = 0.004) and higher follow-up levels of daily-life functioning (left: *β* = 0.30, CI = [0.11, 0.48], *p* = 0.001; right: *β* = 0.20, CI = [0.02, 0.37], *p* = 0.031). A higher baseline gray matter volume in the bilateral insula resulted in more severe positive symptoms at follow-up (left: *β* = 0.22, CI = [0.04, 0.40], *p* = 0.017; right: *β* = 0.20, CI = [0.04, 0.37], *p* = 0.017). A higher somatic health at baseline drove larger gray matter volume in the bilateral cerebellum (left: *β* = 0.09, CI = [0.01, 0.18], *p* = 0.038; right: *β* = 0.15, CI = [0.03, 0.26], *p* = 0.013), while a higher working memory performance (left: *β* = 0.10, CI = [0.02, 0.17], *p* = 0.015; right: *β* = 0.09, CI = [0.01, 0.17], *p* = 0.024) and daily-life functioning (left: *β* = 0.14, CI = [0.02, 0.26], *p* = 0.021; right: *β* = 0.14, CI = [0.02, 0.26], *p* = 0.028) at baseline resulted in larger bilateral gray matter volume in the caudate nucleus. Similarly, a higher working memory performance at baseline predicted larger gray matter volume in the bilateral putamen (left: *β* = 0.13, CI = [0.01, 0.24], *p* = 0.029; right: *β* = 0.14, CI = [0.00, 0.27], *p* = 0.045). No other brain regions revealed similar effects across both hemispheres. A higher positive symptom severity at baseline resulted in worse somatic health at follow-up across all 28 models (for model with global gray matter volume as a neural entity: *β* = −0.25, CI = [−0.06, −0.43], *p* = 0.010). In some models a higher somatic health at baseline was linked to a higher working memory performance at follow-up (Supplementary Table S2), but given the inconsistency of this finding, we did not further interpret it. Supplementary Tables S2 and S3 summarize all statistics, including overall model fits and effect sizes, confidence intervals, and *p*-values for all significant and non-significant paths.

### Mediating role of changes in brain volumes and somatic health

Across all 29 cross-lagged panel models with mediators, only a few significant paths were obtained: all autoregressive paths were significant (Supplementary Table S5 and S7). An increase in global white matter volume from baseline to follow-up drove lower positive symptom severity (*β* = −0.64, CI = [−0.05, −1.23], *p* = 0.033) and higher daily-life functioning (*β* = 0.77, CI = [0.02, 1.51], *p* = 0.044) at follow-up, while a worsening in somatic health was linked to higher daily life functioning (*β* = −0.34, CI = [−0.10, −0.57], *p* = 0.005) at follow-up. A higher working memory performance at baseline resulted in a more pronounced increase of gray matter volume in the bilateral caudate nucleus (left: *β* = 0.09, CI = [0.01, 0.17], *p* = 0.022; right: *β* = 0.09, CI = [0.00, 0.17], *p* = 0.043). No further brain regions showed consistent mediating effects across both hemispheres. Supplementary Tables S4–S7 contain all statistics, including overall model fits and effect sizes, confidence intervals, and *p*-values for all significant and non-significant paths.

## Discussion

Inspired by the long-term aim to identify potential treatment targets in SSD, the current study first explored the reciprocal interrelations over time between symptom severity, cognition, daily-life functioning, brain volumes, and somatic health using longitudinal data from two exercise intervention studies. Further, we investigated whether changes in brain volumes and somatic health mediate the mutual interrelations of clinical symptoms, cognitive performance, and daily-life functioning over time.

### The significance of global Gray matter volume for cognitive outcome in SSD

We found that a lower global gray matter volume at baseline predicted a reduced working memory outcome a few months later. This is in line with the concept of cognitive reserve [49] and corresponds to current findings in the general population, demonstrating that lower initial gray matter volume is linked to more pronounced age-related cognitive decline over time [50]. In people with SSD, global gray matter volume is reduced compared to controls [51]. Our work extends this finding, showing that patients with lower global gray matter volume have worse subsequent working memory outcomes. We did not detect similar effects in other cognitive domains, possibly due to insufficient statistical power. Notably, the effect re-emerged when considering global cognition as the outcome (Supplementary Table S9).

In sum, our results highlight the importance of preventing global gray matter volume decline in SSD, as patients with pronounced gray matter loss are less likely to have a favorable cognitive outcome. Therefore, future treatments in SSD should target general brain health. Global combined lifestyle interventions targeting physical activity, diet, sleep, and substance use may be promising candidates to tackle brain health in SSD, as indicated by respective evidence published in recent years [52–57].

### Hippocampal structural decline and its impact on functional outcome in SSD

Our results further suggest that a lower hippocampal gray matter volume at baseline predicts lower daily-life functioning in SSD at follow-up. Structural impairments of the hippocampal formation in SSD have been repeatedly demonstrated [14, 22, 51, 58–63]. With regard to the clinical implications of hippocampal volume decline in SSD, converging evidence reveals associations with long-term deteriorations in daily-life functioning [64]. Our results build on these findings, indicating that patients with lower baseline gray matter volume in the hippocampal formation show lower daily-life functioning after a few months.

To conclude, our findings emphasize the importance of hippocampal health in SSD, impacting the capability of patients to show improvements in daily-life functioning. Consequently, future treatments should aim to stimulate hippocampal neuroplasticity to ameliorate the global functional outcome of patients. Aerobic exercise interventions [52] or – possibly at some future stage – subcortical brain stimulation methods such as focused ultrasound [65] may reflect promising candidates for such a treatment approach.

## The involvement of default-mode network nodes in disease outcome

Our findings further indicate that a lower gray matter volume in the precuneus at baseline explains higher negative symptoms, lower working memory performance, and worse somatic health at follow-up, while a lower gray matter volume in the posterior cingulate gyrus predicts lower long-term memory performance and daily-life functioning. Both the precuneus and the posterior cingulate gyrus are part of the default-mode network, reflecting a self-referential introspective neural state linked to theory of mind and social cognition [66, 67]. In SSD, cerebral abnormalities in the default-mode network align with more severe negative symptom severity [68, 69], and a decline in working memory [70]. Furthermore, gray matter volume of the posterior cingulate gyrus has been shown to predict functional outcome in SSD [71]. Our findings align with these results, emphasizing the crucial role of the default-mode network in the pathophysiology of SSD. Future studies should evaluate possibilities to influence the structure and function of the default-mode network to improve the overall psychiatric health status of post-acute patients with SSD. Different types of neurostimulation methods could reflect promising candidates [65, 72].

## The impact of white matter volume decline on worse symptomatic and functional outcome

Our findings suggest that decreases in global white matter volume from baseline to follow-up drive subsequent higher positive symptom severity and lower daily-life functioning. White matter pathology in SSD has been mostly associated with cognitive deficits, both on the empirical [23] and the theoretical level [73]. Our data rather point toward its relevance for positive symptom severity and global functioning, but especially the latter strongly depends on cognitive functioning [74]. Hence, strengthening white matter integrity in SSD can be regarded as a promising treatment approach to achieve further global improvements in symptomatology and daily-life functioning. A recent theory suggests a deficient maturation of oligodendrocyte precursor cells to oligodendrocytes as a mechanism causing cognitive deficits in SSD [73]. Based on this idea, a combined therapy of aerobic exercise and clemastine to improve myelin plasticity and global structural connectivity is proposed [73]. Our findings support the clinical relevance of strengthening white matter in SSD.

### Limitations and future directions

Our study includes several limitations that have important implications for future research: First, we aimed to conduct a global exploratory analysis to generate hypotheses regarding the mutual interrelations between clinical outcomes, brain volumes, and somatic health, and thus did not correct for multiple comparisons. Hence, our findings require a replication in an independent sample to evaluate if the identified mechanisms of action are stable across samples. Future longitudinal imaging studies in SSD should use hypothesis-driven approaches, focusing on the relevant regions identified in the current work.

Second, given the limited sample size and our focus on post-acute patients with SSD, the generalizability of our findings remains to be determined. Hence, replication is required in other patient populations to evaluate if the current findings reflect general pathophysiological interrelations or are rather specific at a post-acute disease stage.

Third, we used longitudinal data from two exercise intervention trials in people with SSD without aiming to study exercise-specific effects. Therefore, we also included patients in the control conditions. This approach led to a study sample characterized by differences in exercise intervention type and length, comparable to an observational longitudinal study in which patients may undergo different types of therapies unrelated to the study purpose. However, we acknowledge that the generalizability of our findings may be limited, as our observational sample – where most patients received exercise therapy – may not reflect typical clinical practice. Thus, the current results require a replication in a classical longitudinal observational study in SSD.

Fourth, the directional effects found in this study need to be interpreted with caution. Even though we used cross-lagged panel modeling to study reciprocal effects, there may have been confounding variables unavailable in the current datasets, such as medication change, that may explain the effects obtained. The inverse association between worsening in somatic health and better daily-life functioning may serve as an example, as it contradicts current literature [30], but may be explainable by medication changes during study participation. There may have been changes in antipsychotic treatment for some patients, leading to a worsening of somatic health (e.g., weight gain) accompanied by symptom improvements that also affect the GAF scale. As data on medication change was not available for all patients, we could not test this assumption. Future studies need to repeat the current analyses using a randomized-controlled design while drawing on a larger sample to enable robust causal inferences.

Lastly, using the PANSS negative symptom subscale and the GAF to assess negative symptom severity and daily-life functioning, respectively, reflects a noticeable shortcoming. The PANSS negative symptom subscale also includes items on disorganization and cognition, while the GAF considers overall symptom severity and functioning. Future studies should thus consider more sophisticated assessment methods such as the Brief Negative Symptom Scale [75] or the Functional Remission of General Schizophrenia scale [76].

## Conclusion

Our results suggest that increasing the global gray and white matter volume and the regional gray matter volumes in the hippocampal formation, precuneus, and posterior cingulate gyrus reflect potential treatment targets to achieve further clinical improvements in post-acute SSD. Multiple treatment approaches are promising candidates to address these targets, although a replication of the present findings in independent cohorts is warranted before administering respective interventions in patient populations.

## Supporting information

10.1192/j.eurpsy.2025.10129.sm001 Roell et al. supplementary material Roell et al. supplementary material

## Data Availability

Data can be made available upon request to the corresponding author LR.

## Supplementary material

The supplementary material for this article can be found at http://doi.org/10.1192/j.eurpsy.2025.10129.
